# Acquired diaphragmatic hernia in pediatrics after living donor liver transplantation: Three cases report and review of literature

**DOI:** 10.1097/MD.0000000000010346

**Published:** 2018-04-13

**Authors:** Kai Wang, Wei Gao, Nan Ma, Xing-Chu Meng, Wei Zhang, Chao Sun, Chong Dong, Bin Wu

**Affiliations:** Key Laboratory of Organ Transplantation of Tianjin, Department of Transplant Surgery, Tianjin First Center Hospital, Tianjin, China.

**Keywords:** diaphragmatic hernia, living donor liver transplantation, pediatric

## Abstract

**Rationale:**

Diaphragmatic hernia (DH) in pediatrics following living donor liver transplantation (LDLT) has been seldom reported in the past.

**Patient concerns:**

We report successful diagnosis and treatment of three pediatric cases with DH secondary to LDLT, discuss the possible etiology, and review the relevant literature.

**Diagnoses:**

The primary disease was biliary atresia and DH was diagnosed by computed tomography scan or x-ray of chest.

**Interventions:**

Laparotomy was performed successfully to repair the DH.

**Outcomes:**

The respiratory and digestive function was gradually recovered in 1 to 2 weeks after repair operation. In 2 to 8 months follow-up, patients were asymptomatic without any respiratory or digestive complications.

**Lessons:**

DH post-LDLT should be recognized as a possible complication when a left lateral segment graft is used. Careful clinical examination and prompt surgery could minimize complications.

## 1 Introduction

Since donor shortage became more severe for patients with end-stage liver diseases (ESLD), liver transplantation (LT) with partial grafts, including living donor liver transplantation (LDLT) and split LT, is being paid more attention, due to its advantage of significantly shortening waiting time.^[1]^ Diaphragmatic hernia (DH) is one of the rare complications occurred in pediatric LT, accompanying with multiple patho-physiological factors.^[2]^ DH is usually urgent and requires speedy intervention by surgical treatment. From August 2000 to April 2017, 680 pediatric patients have received LT in our center, and more than half received LDLT. Among which, 3 patients suffered from DH. We, here, report successful diagnosis and treatment of the 3 pediatric cases with DH secondary to LDLT and review the literatures to investigate and analyze the possible etiology.

## 2 Cases report

In total 680 pediatric patients, 471 patients received partial graft LT, in which 407 patients received LDLT and 64 patients received split LT. In pediatric LDLT, 3 cases were diagnosed of DH. The primary diagnosis in these children were biliary atresia (BA) and all of them were performed LDLT at the age of <1 (Table 1). Two of the 3 patients received Kasai operations pre-LDLT. Case 1, 2, and 3 were 9-month-old male, 6-month-old female, and 2-year-old male, who received LDLT from father, mother, and father at 6-month-old, 5-month-old, and 7-month-old, respectively. All LDLT procedures and the postoperative courses were uneventful.

**Table 1:**
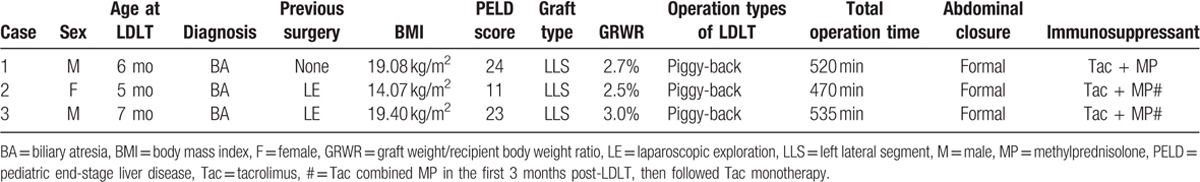
Demographic data of patients in DH pre-, intra-, and post-LDLT.

All subjects signed the Informed Consent Form. The study protocol was approved by the Ethics Committee of the hospital and the study was also conducted in accordance with the principles delineated in the Declaration of Helsinki.

The operative modes of LDLT were piggy-back and the donor grafts types were left lateral segment (LLS). In the construction of outflow tract, the hepatic veins of recipients were modified into triangular type anastomosed to left hepatic vein of donor grafts. The hepatic artery was anastomosed with end to end, and the portal vein of recipients was beveled and interruptedly anastomosed to grafts’ portal vein end to end. Roux-en-Y hepaticojejunostomy was performed, no matter whether the recipients had received Kasai operation pre-LDLT or not.

Immunosuppression regimen was tacrolimus (Tac) combined with methylprednisolone (MP) in the first 3 months post-LDLT, then followed by Tac mono-therapy. No complications were found prior to DH.

Three pediatric recipients were diagnosed with DH post-LDLT (Table 2). Case 1 and case 2 were diagnosed with DH in <3 months, whereas DH was found in case 3 at 16 months post-LDLT. The symptoms of DH in case 1 were mainly respiratory distress with emergent cyanosis. In the early stage of DH, the degree of blood oxygen saturation was down to 70%, and could be relieved by oxygen inhalation. However, respiratory distress and cyanosis symptom re-appeared without oxygen therapy. Right DH was confirmed by urgent chest computed tomography (CT) scan. Other symptom in case 1 included mild dyspepsia for >2 weeks. The symptom of DH in case 2 was early stage mild dyspepsia. The patient was diagnosed as reversed stomach by chest x-ray at the beginning and conservative therapy was adopted for several months. Twelve months later, gastrointestinal symptoms deteriorated, accompanied by anepithymia and vomiting, left DH was then diagnosed in the operation. Case 3 recovered successfully in the following year post-LDLT but presented symptoms of digestive obstruction, such as high fever, frequent vomiting, grievous abdominal distention, and pain. When patient was admitted to hospital, CT scan showed right DH.

**Table 2:**
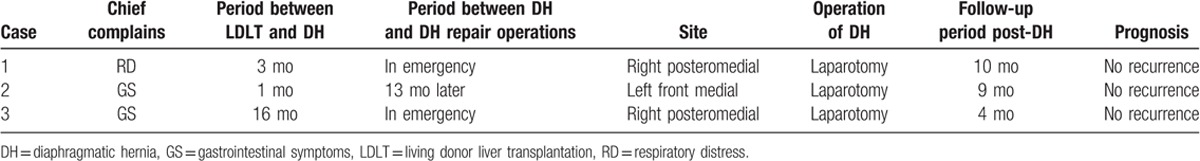
Clinical characteristics of pediatric patients related to DH.

**Figure 1:**
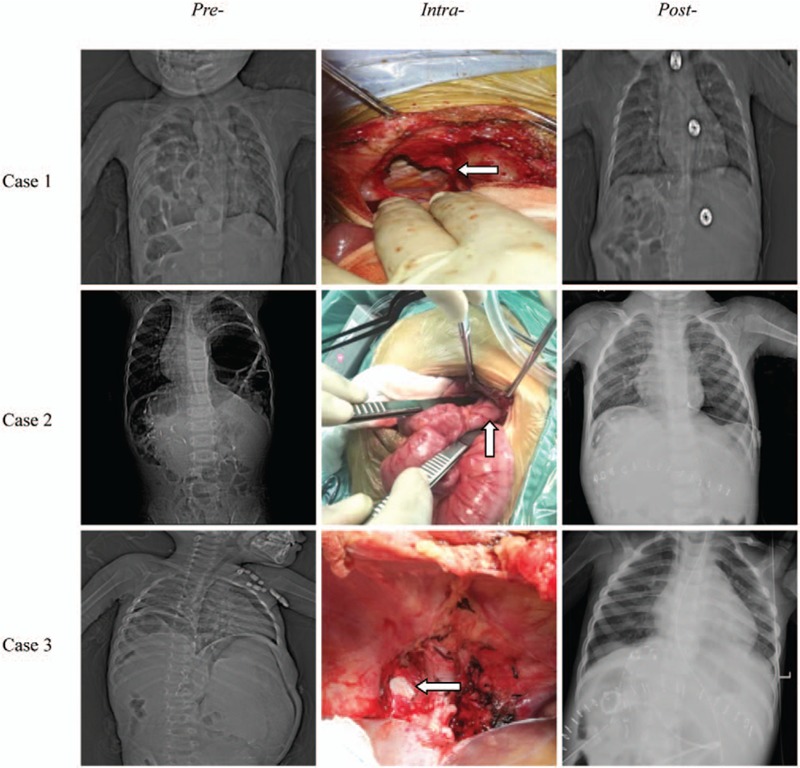
Chest computed tomography scan or x-ray of the patients pre-, intra-, and post-repair operation of DH (case 1 and case 3 were both right DH and case 2 was left DH). 1. Arrows head the position of DH intra-operation. 2. Words on the top of the columns means the time of images. 3. Words on the left of the rows means the order of patients.

Three pediatric patients were performed DH repair operations followed by chest CT scan or x-ray diagnosis (Fig. 1). All surgical repairs were performed by laparotomy along the incision of LT. A 2 cm × 2 cm and a 2 cm × 3 cm post-medial defect were found in the right diaphragm in case 1 and case 3, respectively. In case 2, a 2 cm × 2 cm front medial defect was found in the left diaphragm. Defect in case 1 was extended laterally to return the herniated content back into abdomen, while the herniated contents in case 2 and case 3 were easily returned without extending the defect. Herniated contents included partial intestines among all 3 pediatric patients and partial colon in case 1, proximal stomach in case 2, distal stomach and intestinal loop in case 3, respectively. In case 1 and case 2, the bowel looked viable, however, the herniated intestinal loop in case 3 showed obvious sign of ischemic damage, and Roux-en-Y hepaticojejunostomy was reperformed. Defects in all cases were closed by primary closure without mattresses. The 2 right DH patients were each inserted with a chest tube, whereas no tube was placed in the left DH patient.

Postoperative courses were uneventful, and respiratory and digestive function was gradually recovered in 1 to 2 weeks post repair operation for all 3 cases. At 2 to 8 months follow-up, patients were asymptomatic, without any respiratory or digestive complications.

## 3 Discussion

The most common surgical complications after pediatric LDLT are biliary stricture, biliary leakage, portal vein complications, and hepatic artery thrombosis.^[3]^ DH post-LT is very rare, however, was mostly found in pediatric recipient. Close to 30 cases (27 cases) of DH post-LT in pediatrics have been reported in recent 10 years (Table 3).^[2,4–11]^ All reported patients received grafts on the left side, including left lateral segment (92.6%) and left lobe (7.4%). As to the site of DH, it was more frequent to be on the right side (25 cases, 88%), compared with only 1 case on the left side and 1 case on both sides. In our hospital, the morbidity of pediatric DH post-LDLT was 0.74% (3/407), which is similar to that in other transplant centers. Although the incidence of DH is lower than other complications, the effect of DH can be dramatic and should be paid more attention and be disposed in emergency.

**Table 3:**
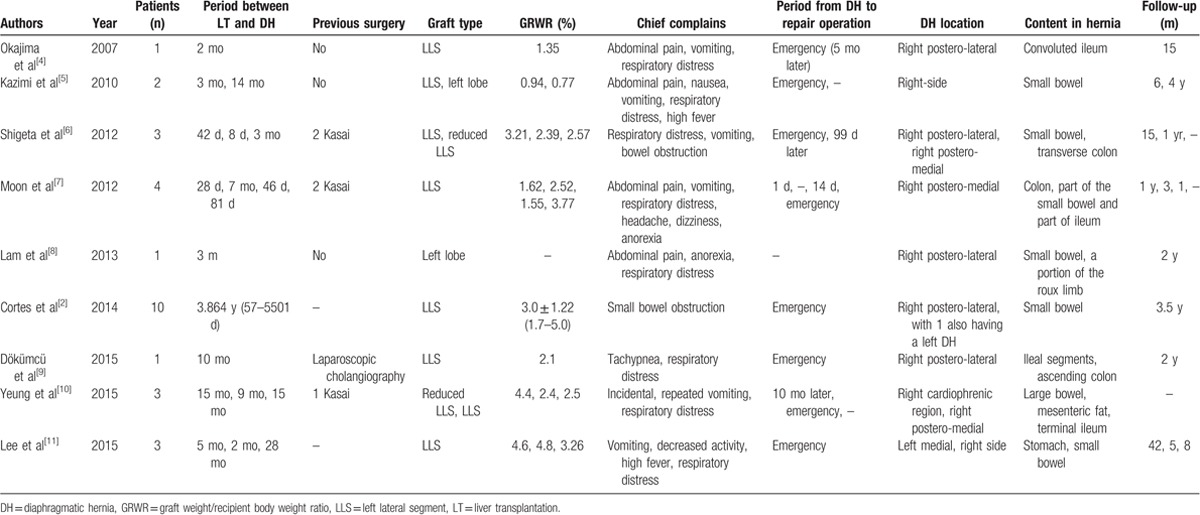
DH in pediatric recipients post-LT reported in literature.

The potential risk factors of DH post-pediatric LDLT include surgical trauma during hepatectomy, condition of left lateral grafts, increased pressure of intra-abdomen, thin musculature of the diaphragm, malnutrition, and immunosuppressants administration.^[12]^ Receiving partial grafts is common, especially for LLS in pediatric LDLT. Majority of reported DH have been left lobe or LLS transplantation which resulted in right diaphragm being unprotected and susceptible to overextension. Severe adhesion between diaphragm and diseased liver formed by previous Kasai operations or injury of bare area caused by dissection might have led to delayed necrosis and perforation.^[6,13]^ Similarly, DH occurred in some cases which were only performed with hepatectomy without LT.^[14,15]^ Furthermore, pediatric recipients were malnourished in general, mostly suffered with hyperbilirubinemia, and were more prone to be injured with delayed healing. It has been confirmed that immunosuppressants, such as steroid or macrolide immunosuppressants, could impair healing post-LT,^[16]^ and Rossetto et al^[17]^ had reported DH post-LT was related with mammalian target of rapamycin. To our experience, surgical trauma in hepatectomy, especially to the bare area, and malnutrition in perioperative period were the main causes of DH.

The most common complaints in DH of pediatric recipients post-LT were respiratory distress, as well as symptoms of gastrointestinal obstruction, including abdominal pain, nausea, and vomiting.^[2,4–11]^ However, DH was not found until respiratory distress or gastrointestinal obstruction. So it was speculated that the trauma of diaphragm happened more earlier than hernia itself in which the hernia hole was enough to afford gastro-intestine through it. In our data, the diameter of hernia hole was 2 cm at least. Earl et al^[18]^ reported an incidence of small bowel obstruction of 3.2% after orthotopic LT in the pediatric population, with diaphragmatic hernias being responsible for nearly half of the cases and lymphoproliferative disease for the remainder. Besides patients’ statements, diagnosis of DH is usually confirmed by chest x-ray or CT scan. CT scan can also accurately detect any major vasculature and adhesions in abdomen post-LT.^[9]^

As to the therapy of DH, it has been agreed that once DH is identified, operation should be performed immediately. Delayed treatment could result in disorder of cardiac and pulmonary function, loss of bowel, and/or severe abdominal infection. Laparotomy was the routine therapy. In 2015, Lee et al and Yeung et al had reported 3 cases of DH, and 1 case was performed by thoracoscopy.^[10,11]^ The courses of DH after surgical repairing were mostly uneventful without any surgical complications. Our experience showed that pediatric patients with DH should receive operation in time, even if the symptoms related to DH are mild. As to the approach of operative intervention, we suggest laparotomy to be the primary option, considering possible obstruction of digestive tract and likely adhesions of abdominal tissues post-LDLT.

## 4 Conclusions

DH post-LDLT is unusual, however, should be recognized as a possible complication when a left lateral segment graft is used. In some cases, DH is found acutely and can even be life-threatening. Diagnosis can easily be made with chest x-ray or CT scan. A high index of suspicion and prompt surgery could minimize complications. When unexplained respiratory or gastrointestinal symptoms after LDLT occurred in pediatric patients, DH should be highlighted in differential diagnosis.

## Acknowledgments

The authors thank Dr Haohao Li for providing photographs of repairing operations of DH.

## Author contributions

Wei Gao proposed the study. Kai Wang performed research, analyzed the data, and wrote the first draft. Nan Ma, Xing-Chu Meng, Wei Zhang, Chao Sun, Chong Dong, Bin Wu helped to collect the data. All authors contributed to the performance of LDLT, the repairing operations of DH, the design and interpretation of the study and to further drafts. Wei Gao is the guarantor.

**Investigation:** B. Wu, C. Sun, C. Dong, W. Zhang, X-C. Meng.

**Methodology:** B. Wu, C. Sun, C. Dong, N. Ma, W. Zhang, X-C. Meng.

**Project administration:** B. Wu, C. Sun, C. Dong, K. Wang, N. Ma, W. Zhang, X-C. Meng.

**Supervision:** W. Gao.

**Writing - original draft:** K. Wang.
